# Oral manifestations in SARS-CoV-2 infection

**DOI:** 10.4317/medoral.25259

**Published:** 2022-06-19

**Authors:** Gulay Tuter, Mukaddes Yerebakan, Bulent Celik, Gulsah Kara

**Affiliations:** 1 Professor, Department of Periodontology, Faculty of Dentistry, Gazi University, Ankara, Turkey; 2 Research assistant at Gazi University Faculty of Dentistry Department of Periodontology Ankara, Turkey; 3 Professor, Gazi University Faculty of Science Department of Statistic, Ankara, Turkey; 4 Research assistant at Gazi University Faculty of Dentistry Department of Periodontology Ankara, Turkey

## Abstract

**Background:**

We aimed to evaluate the prevalence of predisposing factors and oral manifestations in SARS-CoV-2 infection.

**Material and Methods:**

204 SARS-CoV-2 positive patients were included in the study. Questions regarding the systemic, periodontal health, oral hygiene habits, common symptoms and, oral manifestations of COVID-19 such as oral lesions, and dry mouth were included in the survey. The Visual Analogue Scale (VAS) was used.

**Results:**

47.5% of individuals had various systemic diseases. Dry mouth (44.2%) and oral lesions (22.4%) were the most common oral manifestations in COVID-19 patients. Also, dry mouth had the highest VAS score. The most common oral lesion locations were buccal mucosa (15.2%) and tongue (10.8%). The majority of participants (142 patients) were affected by taste disorders. Patients who received periodontal treatment before SARS-CoV-2 infection reported fewer oral complaint and manifestations than those who did not receive periodontal therapy (*p*=0.032). There was no statistically significant difference between males and females on the presence of any oral manifestations, and taste disorders.

**Conclusions:**

Our results showed that SARS-CoV-2 could cause oral manifestations. However various predisposing factors may be part of the etiology and promote oral findings.

** Key words:**SARS-CoV-2, COVID-19, xrestomia, dysgeusia, oral manifestation.

## Introduction

The novel coronavirus, severe acute respiratory syndrome coronavirus 2 (SARS-CoV 2) belongs to the Coronaviridae family and it is the cause of coronavirus disease 2019 (COVID-19). This novel strain SARS-CoV-2 has caused a pandemic declared by The World Health Organization (WHO) in March 2020 (1).

Current knowledge about the primarily transmission routes of SARS-CoV-2 defines a direct transmission via droplets as caused by sneezing, talking, coughing and, a contact transmission by contact with the mucous membranes such as nasal, ocular and, oral. Also, there might be other routes of transmission through the fetal-oral and the saliva (2). It has been reported that the receptor-binding domain of the SARS-CoV-2 S-protein has a strong affinity binding to the angiotensin-converting enzyme 2 (ACE2) (3). Therefore, organs with ACE2 expressing cells can be the main target for SARS-CoV-2. ACE2 expression was detected in the cell membrane of various tissues and organs such as liver, kidneys, lungs, upper respiratory tract, nervous system and epithelial cells of oral mucosa including tongue and salivary glands (3,4).

COVID-19 patients may experience different clinical signs and symptoms in multiple organs of the body. These symptoms can be within a range from asymptomatic condition to acute respiratory distress syndrome, multi-organ dysfunction and, even death. COVID-19 patients may present main symptoms such as fever, dry cough, tiredness, headache, diarrhea and conjunctivitis in various severity (5). In addition to main symptoms, some oral manifestations as xerostomia, aphthous like-lesions, ulcers, tongue depapillation, necrotising gingivitis, taste disorders and, salivary gland infections have also been reported in SARS-CoV-2 infection (6,7). Furthermore, SARS-CoV-2 was detected in saliva of COVID-19 patients (8). Because of the oral cavity can be the entry for pathogens, it can be suggested that SARS-CoV-2 has oral symptoms, as in many other viral infections (9). Therefore, we aimed to evaluate the prevalence of predisposing factors and oral symptoms-manifestations of SARS-CoV-2 infection by a questionnaire survey carried out by COVID-19 patients in this study.

## Material and Methods

- Study participants

The Ethics Committee of the Faculty of Dentistry, Gazi University approved the study according to the Helsinki Declaration of 1975, which was revised in 2013 (Protocol ID: E-21071282-050.99-38278). The present study was also assessed and approved by Ministry of Health of Turkey (2021-01-05T12_31_45). The online survey titled ‘Evaluation of oral manifestations in SARS-CoV-2 positive patients’ was created by online platform of Google Forms (Google, LLC). The form has been designed in such a way that it can be easily filled in a short time. The questionnaire was sent to 220 SARS-CoV-2 positive patients whose contact information was obtained from The Isolation and Tracking System controlled by Hospitals of Ministry of Health database. Firstly, the investigators (M.Y., G.K.) contacted to COVID-19 patients and gave information about our study on the phone. Based on their verbal consent, an online questionnaire link was sent to each patient’s mobile phone. A digital consent was also obtained before responding to the survey. Every participant was able to submit the questionnaire only once. 6 patients who had advanced age gave their verbal consent and asked to respond to the questionnaire by talking to the investigator instead of filling out the form. The investigator filled out the survey as in line with their answers and the data was transferred to our study questionnaire through Google Forms.

SARS-CoV-2 positive patients (confirmed by polymerase chain reaction -PCR-) over 18 years of age, of any gender who wish to participate in the study were included in the present study. There were no exclusion criteria. 16 COVID-19 patients who did not want to participate in the survey were excluded.

- Questionnaire

An online survey was carried out from February 2021 to March 2021 (Table 1, Questionnaire). The survey questions included choices but some questions had additional options in which the patients could describe their problems if the given options were not applicable to them. The questionnaire included 5 sections and 35 questions. A brief introduction about the study including the aim of the study and the consent section was available prior to the survey. Demographic data, systemic health and smoking status and, regular medication use were included in section 1. There were questions related with oral hygiene habits and preventive oral care behaviors before and during COVID-19 infection in the second section. Common symptoms of COVID-19 such as fever, cough, sore throat and, information about the treatment period such as hospitalized or taken home care and, drugs used were questioned in section 3. In addition that, oral manifestations such as pain, bleeding, dry mouth, swelling, presence of any lesion, change in taste sensation were asked in section 3. Also, any dental therapy need of patients was questioned while being infected by SARS-CoV-2 in section 3. Section 4 contained questions related to the participant’s periodontal status and periodontal treatment history to determine whether periodontal diseases were associated with oral symptoms of COVID-19. The Visual Analogue Scale (VAS) was used to assess tooth pain, gingival pain, gingival bleeding and, dry mouth in section 5. The information about the VAS was provided and stated that "the number 0" was the least and "the number 10" was the most effective situation. Subjects were asked to give a score between 0 and 10 for these variables. Taste dysfunction duration was also asked in section 5.

- Statistical Analysis

Prior the study, a power analysis was performed using G*Power Software (v3.1.3; Franz Faul, Universität Kiel, Germany) to determine the appropriate sample size required for the study. With a significance level of 0.05, small effect size and a power of 95%, it was determined that 204 patients should be included in the study.

All statistical analyses were performed using Statistical Package for Social Sciences (SPSS) version 15 software (SPSS Inc., Chicago, IL, USA). Continuous variables were presented as mean and standard deviation values whereas categorical variables as frequencies and percentages. Independent two groups were compared with the Independent samples t-test for continous variables. The relationships between categorical variables were evaluated with Pearson’s Chi-Square (or Fisher’s exact test when appropriate) and Cramer’s V statistics. A two-sided *p* value less than 0.05 was considered statistically significant for all analyses.

**Table 1:**
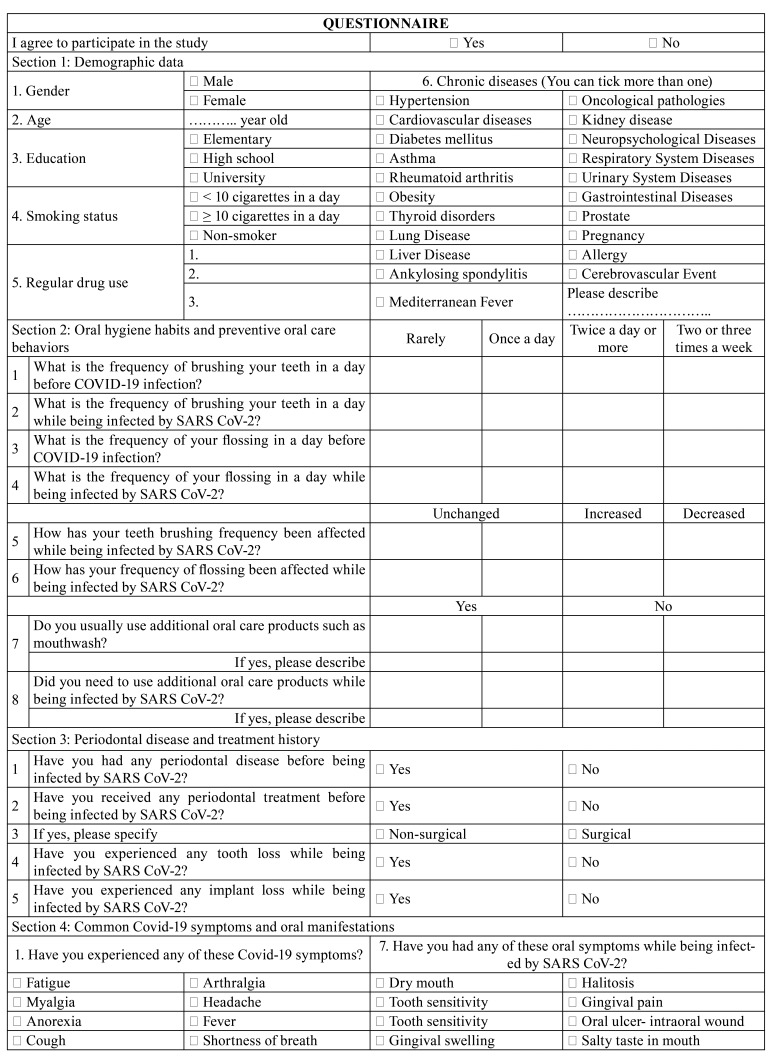
Questionnaire.

**Table 1 cont.**
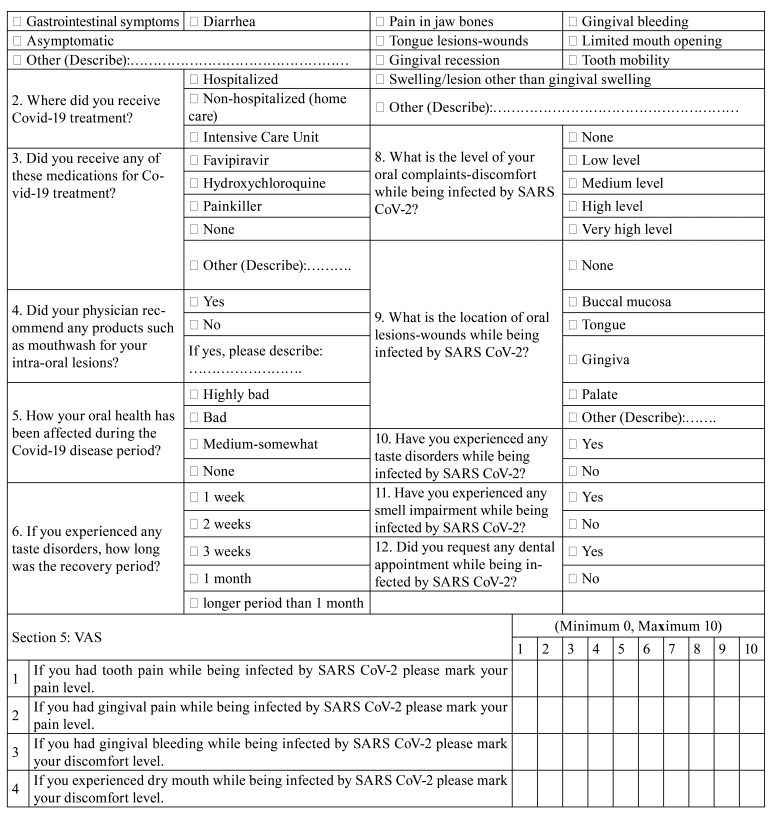
Questionnaire.

## Results

204 voluntarily participants who diagnosed COVID-19 were included into the study. None of the study patients were vaccinated. There was no statistically significant difference between males (mean±SD, 53,3±17,8) and females (48,6±19,1) in the meaning of age (*p*=0.083). Regarding the educational level, participants were evaluated as follows: elementary and high school (59.3%) and, university education (40.7%). Demographic distribution of study subjects are shown in Table 2. The majority of individuals (42.6%) were between 31 and 60 years old. Our results showed that 47.5% of COVID-19 patients had systemic diseases and 47.1% of them were under regular drug treatment. Moreover, the most prevalent chronic diseases were hypertension (62.9%), cardiovascular diseases (59.8%) and diabetes mellitus (34.0%). In addition, as we categorized ‘other’, 2.1% of patients were affected by lung disease, 3.1% of patients were affected by liver disease, and 3.1% of patients were affected by oncological pathologies. One of the study group subjects was pregnant (1.0%). The majority of the study participants were non-smokers (85.3 %) (Table 2).

**Table 2:**
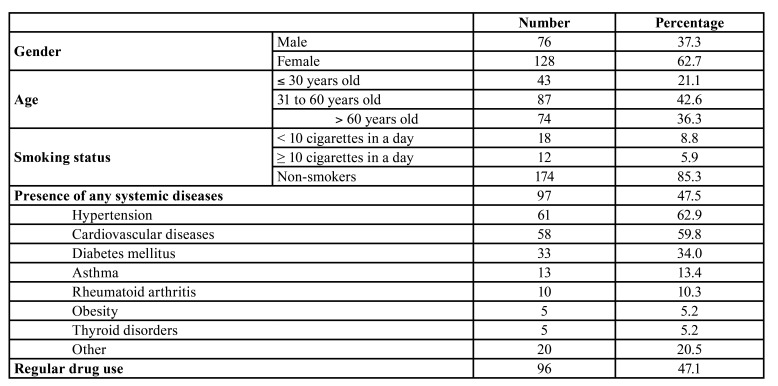
Demographic distributions of COVID-19 patients.

Regarding the common symptoms of COVID-19, most of the patients had one or more symptoms such as fatigue (78.9%), arthralgia (69.6%), myalgia (59.3%) but there were also asymptomatic patients (5.4% without any symptom). It was detected that women were worse affected than men by some of the COVID-19 symptoms. There were statistically significant differences between women and men on the presences of fatigue, arthralgia, headache and gastrointestinal symptoms (*p*< 0.05).

Our results showed that 81.9% of patients were non-hospitalized (medication use/home care) during their COVID-19 infection period. However, 16.2% of patients were hospitalized and 2.0% of them were treated in the intensive care unit. The study participants reported that they used prescribed drugs such as Favipiravir (68.1%), hydroxychloroquine (31.9%) and, pain killer (2.0%). However, there were patients who did not use any drug (21.1%). It was detected that any prescription was not given to study participants (92.6%) related with their oral health in this study.

According to our results, most of the patients brushed their teeth once (37.7%) or twice a day (28.9%) as it was before while being infected with SARS CoV-2. However, 11.3% of the study participants reported that their brushing frequency decreased. Similarly, our findings showed that the number of patients who used regular dental floss decreased (7.4%). Furthermore, 20.6% of participants reported that they rarely brushed their teeth while being infected with SARS-CoV 2. Our results showed that 41.2% of patients did not need to use any additional mouth wash before the infection. However, 35.8% of participants preferred to use an addional mouth wash or saltywater (22.5%).

Regarding periodontal disease and periodontal therapy history, 39 patients reported that they had periodontal disease and received non-surgical periodontal therapy (13.7%) and periodontal surgery (5.4%). However, the majority of participants reported that they received neither non-surgical periodontal therapy (86.3%) nor periodontal surgery (94.6%) before COVID-19 infection.

33.3% of patients reported that their oral health was not affected by COVID-19 in this study. However, some of the study participants reported that their oral health was affected as follows: highly bad (10.8%), bad (21.1%) or medium-somewhat (34.8%).

Our results showed that dry mouth (xerostomia) was the most common oral manifestation (44.2%) in COVID-19 patients (Table 3). The second common oral manifestations were oral lesions (22.4%) such as oral ulcer (14.5%) tongue lesions (6.7%) and lesion other than gingival swelling (1.2%). There were no statistically significant differences between males and females on the presences of any oral manifestations (Table 3). Some patients reported that they had dental filling fracture, increased staining on teeth and, increased calculus formation which we categorized as ‘other’ into Table 3. Regarding oral discomfort related with oral manifestations, it was determined that 19.1% of the participants did not have any oral complaints. But there were 10 patients (4.9%) with very high discomfort level and 14 patients (6.9 %) with a high discomfort level. Some study participants reported that they lost teeth (6 patients, 2.9%) and dental implants (3 patients, 1.5%). Our findings showed that the majority of study participants (86.3%) did not request any dental appointment while being infected by SARS-CoV 2. As a most common oral manifestation, dry mouth did not show any significant difference between males and females in this study (*p*=0.627). Similarly, dry mouth was not statistically different among patients in different age groups (*p*=0.549). However, dry mouth had the highest VAS score with a mean VAS score of 4 in this study (Table 4).

One hundred-forty three patients (70.1%) reported that they had no oral lesion during the infection period. However, 61 patients reported that they had oral lesions located in one or more different oral areas. It was determined that the most common oral lesion location was buccal mucosa (15.2%). Tongue was the second oral lesion area (10.8%) in the study patients. Gingiva and palate were the other oral lesion sites with the same percentage (8.8%).

Our results showed that the most of participants (142 patients) were affected by taste disorders in this study. 71.9% of women and 65.8% of men reported that they had taste disorders. Regarding taste changes, there was no statistically significant difference between males and females in this study (*p*=0.361). Similarly, taste disorders did not show any significant difference among patients in different age groups (*p*=0.129). COVID-19 patients reported that the recovery duration of taste disorders was 1 week (29.6%), 2 weeks (26.1%), 3 weeks (11.3%), 1 month (10.6%) and, longer period than 1 month (22.5%). In addition to taste disorders, 65.7% of COVID-19 patients (134 participants) had smell impairment in this study.

Regarding to the prevalence of oral manifestations in relation to the patient’s periodontal therapy history, it was found that there was a correlation between those who recieved periodontal therapy before COVID 19 infection period and those who did not (*p*=0.032, Cramer’s v=0.227). The study participants who recieved periodontal treatment prior COVID-19 infection had less oral complaint and oral manifestation than those who did not receive periodontal therapy in our study.

**Table 3:**
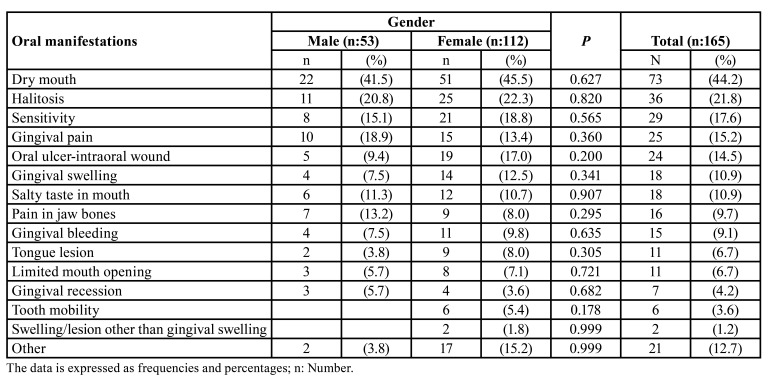
Distribution and comparison of oral manifestations between males and females in the study.

**Table 4:**
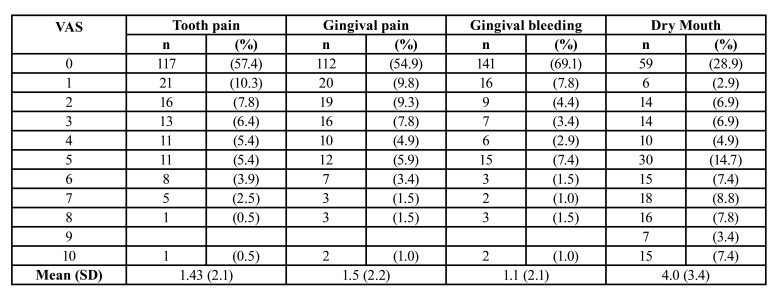
VAS recordings in COVID-19 patients.

## Discussion

It is known that various systemic viral infections like human immune deficiency virus have oral lesions (9). The association between oral manifestations and COVID-19 remains an important issue to be clarified as the COVID-19 pandemic continues. Therefore, in this study, we aimed to evaluate the prevalence of predisposing factors and oral manifestations in patients while being infected by SARS-CoV-2.

Regarding the presence of any systemic diseases, our results showed that 47.5% of COVID-19 patients had systemic diseases. In a previous study, comorbidities have been evaluated in 5700 patients with COVID-19. Authors stated that the most common comorbidity was hypertension in COVID-19 patients (10). In agreement with their data, another observational study reported that most of the COVID-19 patients had hypertension (11). Our results were similar with these previous findings (10,11). In fact, 62.9% of COVID-19 patients had hypertension in our study. According to the data in the literature, it can be suggested that the severity risk of COVID-19 is increased by hypertension. However, it is not clear whether hypertension is a risk factor being infected by SARS-CoV-2. As an interesting finding, the majority of participants (85.3%) were non-smokers in the present study. Parallel with our results, Sinjari *et al*. showed that approximately 90% of COVID-19 patients were non-smokers in their study (11). Likely, in a multicenter European survey, it was found that 86.6% of COVID-19 patients were non-smokers (5). It can be suggested that smoking is associated with the severity of COVID-19 but there is no clear evidence whether smokers are in a high infection risk by SARS-CoV-2.

According to our results, during the COVID-19 infection period, the most of patients (66.6%) brushed their teeth once or twice a day as it was before. However, other participants reported that their brushing frequency decreased while being infected with SARS-CoV-2. Similarly, in an observational study, the number of patients who brushed three times a day decreased in COVID-19 infection period (11). The majority of participants reported that they had received neither non-surgical nor surgical periodontal therapy in our study. However, 39 patients reported that they had periodontal disease history and received either non-surgical periodontal therapy or periodontal surgery before COVID-19 infection. It is known that periodontal pockets are a harbor for subgingival biofilms and interact with the soft tissue wall of the pocket and the oral cavity (12). Also, some viral species as the Herpes simplex viruses are found in periodontal pockets. Therefore, in a previous study, it was hypothesized that periodontal pocket could act as a reservoir for SARS-CoV-2(12). Infact, the patients who received periodontal therapy had less oral discomfort and/or oral manifestation than those who did not receive periodontal therapy in our study. It can be suggested that periodontitis may increase the severity of oral manifestations in COVID-19 patients.

In the present study, dry mouth was the most common oral manifestation in COVID-19 patients. Moreover, as parallel with previous study results (13,14), dry mouth did not show any significant difference between males and females and also there was no statistically significant difference among patients in different age groups in our study. A systematic review reported that a high xerostomia ratio was found in COVID-19 patients (15). Chen *et al*. showed that dry mouth was one of the two major oral manifestations in COVID-19 patients in their questionnaire (8). Similarly, it was found that 47.6% of patients with COVID-19 manifested xerostomia in a questionnaire involving 573 participants (14). Moreover, xerostomia was shown as an initial symptom of COVID-19 and 56% of participants reported that they had dry mouth in another survey (13). Likely, it was reported that dry mouth was one of the symptoms of the patients and dry mouth appeared before the other common COVID-19 symptoms (16). However, it should be noted that different drug therapies such as antihypertensives, anticholinergics and antidepressants can cause dry mouth. Furthermore, it has been stated that xerostomia is common in diabetic patients (17). In fact, diabetes mellitus was one of the most prevalent chronic diseases (34.0%) in our study. In addition, our results showed that 47.1% of participants were under regular drug treatment because of their chronic diseases. Also, our study subjects reported that they used prescribed drugs such as Favipiravir (68.1%) and hydroxychloroquine (31.9%) while being infected by SARS-CoV-2. Medical treatments associated with COVID-19 can also lead to xerostomia due to reduced saliva flow (18). Therefore, dry mouth may be either directly associated with SARS-CoV-2 infection or it may be partly associated with drug therapies and chronic diseases in our study.

It has been shown that SARS-CoV-2 binds to ACE2 which is the primary receptor of this virus. Cells with ACE2 receptor distribution can be the target cells for the virus due to SARS-CoV-2 enters into these cells via ACE2 (3,7). It was detected that the ACE2 expression was present in various tissues (4). Chen *et al*. confirmed that ACE2 was expressed by salivary glands (8). Also, it was stated that the ACE2 receptor numbers were higher in the salivary glands as compared with the lungs (4). In a clinical study, it was shown that SARS-CoV-2 was detected in saliva samples of 91.7% of patients and the live virus isolated from saliva was confirmed by viral culture (19). It was hypothesized that SARS-CoV-2 could lead to pathological inflammatory lesions in salivary glands; then, SARS-CoV-2 could cause acute sialoadenitis and then saliva secretion decrease. Moreover, secretory dysfunction of the salivary glands may be early symptoms of COVID-19. In fact, in a previous study, acute parotitis was reported as an initial symptom of COVID-19(20). Regarding the etiology of dry mouth in COVID-19 patients, although various factors may be part of the etiology, these findings can suggest that dry mouth may be associated with SARS-CoV-2 infection in COVID-19 patients.

In the present study, oral lesions such as oral ulcers and tongue lesions were the second common oral manifestations in COVID-19 patients. Similarly, Carreras-Presas *et al*. showed that COVID-19 patients had oral mucosal vesiculobullous lesions, painful ulcers and tongue pain in their case report (6). Moreover, Halepas *et al*. reported that COVID-19 caused a postviral immunologic reaction resulting in a multisysytem inflammatory syndrome in children (MIS-C) (21). In their retrospective review of pediatric patients with COVID-19, it was shown that 47 pediatric patients with MIS-C had oral or oropharyngeal manifestations such as red or swollen lips and strawberry tongue. Related with their findings, they concluded that oral manifestations could be an early indicator of MIS-C (21). In another review, oral manifestations associated with COVID-19 were summarized with the data of 25 COVID-19 patients (22). It was reported that COVID-19-associated oral manifestations were highly heterogeneous and the most common ones were oral ulcers, vesiculobullous lesions, acute sialadentitis and parotitis. Furthermore, it was stated that oral lesions could be the initial symptoms in several COVID-19 cases (22). Parallel with our results, Fidan *et al*. reported that 78.4% of COVID-19 patients had oral lesions such as aphthous-like ulcers, erythema and lichen planus in their survey (23).

It has been shown that the ACE2 is highly expressed by epithelial cells in oral mucosa like tongue mucosa (4). It was also stated that there was a higher release of ACE2 in tongue epithelial cells compared to buccal or gingival tissues (6). Related with these findings, it was suggested that the oral cavity could have important roles as being a portal of entrance of SARS-CoV-2 and as a reservoir of the virus (24). Moreover, it has been stated that SARS-CoV-2 could accumulate at the oral, nasal and pharyngeal mucosa after the transmission. In fact, we determined that the most common oral lesion locations were buccal mucosa and tongue in our study. Although it is difficult to assess the cause of these lesions, it can be suggested that oral mucosa may be targeted by SARS-CoV-2, which allows viral replication and may cause tissue inflammation and destruction. Therefore, as it was stated in a previous study, the etiology of oral lesions in COVID-19 patients could be associated with SARS-CoV-2 infection (25). Regarding the etiology of oral lesions in COVID-19 patients, in contrast, a study stated that there was not sufficient evidence to support an oral damage caused by SARS-CoV-2 (26). However, COVID-19 infection, together with co-infections, immunity impairment and adverse drug reactions from medications to COVID-19 treatments can be suggested among the probable causes of oral lesions in COVID- 19 patients (27).

Our study results showed that most of the participants were affected by taste disorders (142 patients) and smell impairment (134 patients). Similarly, in a systematic review, it was reported that there was a positive association between taste disorder symptoms and COVID-19. Furthermore, taste disorders were significantly associated with female patients in that review (27). Different than their report, regarding taste changes, there was no statistically significant difference between males and females in our study. Infact like our findings, taste disorders showed no statistically difference between males and females in a previous study (13). Although taste disorder pathogenesis in COVID-19 patients is not fully understood, Finsterer *et al*. suggested that the possibility of a local inflammatory response resulting from rhinitis trigers, which could hamper the normal function of taste buds (28). In addition, Vaira *et al*. reported that taste disorders may pose as an adverse event by concomitant oldfactory disorder in COVID-19 patients (29). Furthermore, the interaction of SARS-CoV-2 with gustatory components and ACE2 receptors may support a direct effect in COVID-19 related with taste disorders (28). As it was suggested before, SARS-CoV-2 may bind salivary components accelerating taste particle degredation and disturbing gustatory sensation (29).

The clinical examination of COVID-19 patients was not applicable in this study as we could contact to them by the phone and survey. This can be a limitation of our study. However, as we stated in our results, almost none of these COVID-19 patients requested dental appointment while being infected by SARS-CoV-2. This may be because COVID-19 patients concentrate more on their general health problems than oral complaints. Furthermore, as it has been shown in a previous review, most of the studies performed with oral examination were conducted with a few cases (30). Due to this fact, survey studies can provide important results regarding the prevalence of oral manifestations in COVID-19 patients.

It has been stated that cytokine storm caused by humoral and cellular mechanisms may worsen existing autoimmune conditions in the oropharyngeal area(9). In addition, elevated pro-inflammatory mediator levels in COVID-19 patients and oral infections can impair tissue homeostasis (30). Therefore, as it was confirmed by many studies in a recent review,(30) within the limits of our results, it can be suggested that COVID-19 may include oral manifestations and symptoms. However, these manifestations should be investigated in particular due to it still remains unclear whether these manifestations are directly caused by SARS-CoV-2 infection or a secondary outcome caused by the impaired immune system and adverse reactions of medical treatment.

## Conclusions

Although various factors may be part of the etiology, our results showed that SARS-CoV-2 could cause oral manifestations such as dry mouth, taste disorders and buccal mucosa and tongue lesions in COVID-19 patients. Various predisposing factors may promote oral findings. Due to this fact, we suggest that oral hygiene should be improved or at least maintained while being infected with SARS-CoV-2 in order to reduce the bacterial load in the mouth and the risk of a bacterial superinfection. Poor oral hygiene and periodontal disease can be considered as a risk to post-SARS-CoV-2 viral complications and oral manisfestations specialy in patients with chronic diseases. Therefore, dentists should be part of the multidisciplinary team for the diagnosis and treatment of COVID-19 patients. Further studies are needed to clarify whether oral manifestations in COVID-19 patients are directly caused by SARS-CoV-2 or secondary manifestations.
